# Art for health’s sake or health for art’s sake: Disentangling the bidirectional relationships between arts engagement and mental health

**DOI:** 10.1093/pnasnexus/pgae465

**Published:** 2024-10-17

**Authors:** Hei Wan Mak, Yang Hu, Feifei Bu, Jessica K Bone, Daisy Fancourt

**Affiliations:** Department of Behavioural Science and Health, Institute of Epidemiology and Health Care, University College London, 1-19 Torrington Place, London WC1E 7HB, United Kingdom; Department of Sociology, Lancaster University, Lancaster LA1 4YN, United Kingdom; Department of Behavioural Science and Health, Institute of Epidemiology and Health Care, University College London, 1-19 Torrington Place, London WC1E 7HB, United Kingdom; Department of Behavioural Science and Health, Institute of Epidemiology and Health Care, University College London, 1-19 Torrington Place, London WC1E 7HB, United Kingdom; Department of Behavioural Science and Health, Institute of Epidemiology and Health Care, University College London, 1-19 Torrington Place, London WC1E 7HB, United Kingdom

**Keywords:** nonrecursive model, arts engagement, mental health, GHQ-12 mental distress, SF-12 MCS mental well-being

## Abstract

Increasing evidence links arts engagement with mental health, but the directionality of the link remains unclear. Applying a novel approach to causal inference, we used nonrecursive instrumental variable models to analyze two waves of data from the United Kingdom Household Longitudinal Study (*n* = 17,927). Our findings reveal bidirectional causal relationships between arts engagement (arts participation, cultural attendance, and heritage visits) and mental health (GHQ-12 mental distress and SF-12 Mental Component Summary mental well-being). After adjusting for time 1 measures and identifying confounders, cultural attendance and heritage visits were reciprocally associated with mental distress and mental well-being, while arts participation was only reciprocally associated with mental well-being. The bidirectional effects between arts engagement and mental health are modest but clearly demonstrated not only from mental health to arts but also from arts to mental health. Our findings indicate that previous evidence of an association between arts engagement and mental health is due to bidirectional causal effects. Interventions that boost arts participation, cultural attendance, and heritage visits may help break the negative feedback loop and enhance mental health.

**Significance Statement**

This study addresses important theoretical and methodological questions surrounding the directionality of the relationship between engagement with the arts, culture and heritage, and mental health. It shows that this relationship is causally bidirectional. After adjusting for time 1 measures and identifying confounders, cultural attendance and heritage visits are reciprocally associated with mental distress and mental well-being, while arts participation is only reciprocally associated with mental well-being. We also found that the bidirectional causal effects between arts engagement and mental health are similar in size.

## Introduction

Over the past two decades, increasing recognition of the potential of arts activities in helping improve mental health has been transforming attitudes and approaches to mental health care. Current developments include an increasing emphasis on “prescribing” the arts to support the treatment and management of mental health conditions, as well as embedding arts and mental health reciprocally within the strategic objectives of public health and cultural organizations to improve and maintain population mental health (1, 2). Arts engagement broadly encompasses arts participation (e.g. singing, dancing, and acting), cultural attendance (e.g. opera, exhibitions, and galleries), and heritage engagement (e.g. visiting historical sites, parks, and monuments). Despite decades of research demonstrating that arts activities are associated with better mental health (3), the directionality of the association remains unclear.

On the one hand, researchers have used various designs to assess how arts engagement influences multidimensional aspects of mental health. To date, >3,000 studies have identified the role of the arts in helping promote good mental health and prevent and manage mental health conditions. For instance, intervention studies that compare outcomes between treatment and control groups have shown that activities, including singing, visiting museums, group drumming, dancing, photography, drama, drawing, and painting, are associated with improved psychological well-being and reduced depression and anxiety (4–8). These studies are supported by larger scale observational research showing that arts engagement is longitudinally and positively associated with happiness, life satisfaction, self-esteem, resilience, positive effect and purpose in life, and negatively correlated with loneliness and social isolation (3).

In seeking to understand the mechanisms underlying these findings, hundreds of processes have been identified across various fields (9, 10). Psychological research has demonstrated how the arts can help regulate emotions, enhance a sense of self, and support coping and resilience (11). Neuroscientific research has identified the ability of the arts to modulate arousal, engage emotion-related brain networks, activate reward networks (including triggering dopamine release), and release neuropeptides such as oxytocin (12). Psychobiological research has identified reductions in neuroendocrine markers of stress (including catecholamines and glucocorticoids), regulation of inflammatory biomarkers (such as cytokines), and regulation of cardiovascular and electrodermal activity (13). Social science research has shown how the arts can facilitate social bonding, increase social capital, and reduce social isolation, all of which are linked to better mental health (3). Further behavioral processes have also been identified, including those related to the maintenance of healthy lifestyles and health literacy that reduce risk factors associated with mental ill health (10).

On the other hand, however, arts engagement is far from equal. Those with poorer mental health, long-standing mental health conditions, and those experiencing low levels of happiness are less likely to engage in cultural activities (e.g. live music events, opera, exhibitions) (14) and participatory arts (e.g. singing, dancing, arts, and crafts) (15). These associations exist independent of individuals’ sociodemographic characteristics, although it has also been shown that individuals’ socioeconomic position and area deprivation explain part of the association between mental health and engagement rates (14). As a result, the association may reflect differences in individuals’ experience of barriers relating to psychological and physical capabilities (e.g. lack of confidence, lack of sufficient energy, and strength), physical and social opportunities (e.g. limited access and opportunities), and motivations (e.g. less interest) to engage in arts activities (14–16). Furthermore, engagement is lower among groups that are disproportionately more likely to experience poorer mental health (17, 18), including those from racial/ethnic minority backgrounds, with fewer educational qualifications, of lower socioeconomic status, and living in more deprived areas (19–22).

Considered together, the two separate bodies of research imply that the relationship between arts engagement and mental health may be bidirectional or reciprocal, and theories applying the lens of complex adaptive systems science suggest that positive and negative feedback loops may support the maintenance of this bidirectionality (10). However, this hypothesis remains to be tested explicitly. The directionality and magnitude of the link between arts engagement and mental health are important to explore for at least three reasons. First, exploring whether the association is unidirectional or reciprocal could provide important insights into whether a feedback loop does in fact exist. Previous studies have been criticized for assuming bidirectionality in the absence of empirical data. Second, if arts engagement does exert an influence on mental health, this would have implications for the design of public health and policy strategies aiming to promote better mental health through lifestyle and behavioral changes. Third, if mental health does impact subsequent arts engagement, this has implications for cultural organizations working to develop strategies to ensure equitable opportunities for arts engagement, suggesting that specific efforts may be required to ensure accessibility for people with a mental health condition.

Against this backdrop, this study was designed to disentangle the directionality of the relationship between arts engagement and mental health using a nonrecursive structural equation modeling strategy with instrumental variables (IVs). We included three different dimensions of arts engagement (arts participation, cultural attendance, and heritage visits), which involve various active ingredients and mechanisms of action (9, 10), exploring whether and with what level of magnitude a bidirectional relationship can be seen. Further, given that there is a widely accepted two-continuum model of mental illness and mental well-being (such that individuals can experience one but not the other), we considered both dimensions independently (mental distress and mental well-being), to enable a comparison of whether a bidirectional relationship is found for both negative and positive aspects of mental health.

## Methods

### Data

This study used data from the United Kingdom Household Longitudinal Study (UKHLS, www.understandingsociety.ac.uk), which started in 2009 and interviewed around 40,000 households and 50,000 individuals in the baseline wave. The respondents are re-interviewed every year, with new sample members added to compensate for attrition. The UKHLS collects rich longitudinal data on participants’ demographic characteristics, socioeconomic positions, leisure activities, participation in the arts, and health. In our study, data from waves 2 (2010–2012) and 5 (2013–2015) were analyzed—the only waves in which questions on arts engagement were included. Of the 54,569 adult participants (aged 16 and above) in wave 2 (time 1), 42,762 were followed up in wave 5 (time 2). The UKHLS contains a self-completion module, which includes questions on mental health; and only a representative subsample of respondents was invited to take part in the module. Of the respondents who participated in both waves, 27,877 took part in the self-completion module. In total, 26,023 participants provided complete data across all measures. Missing data (6.7%) were handled using listwise deletion. Little's test of missing completely at random (MCAR) showed that the missing data were not MCAR (χ^2^ distance = 5,353.5, degree of freedom = 758, *P* < 0.001) (23). The missingness was largely attributed to responses on whether or not respondents had been clinically diagnosed with depression, education level, and living area. We used the longitudinal self-completion weights developed by the UKHLS team to account for survey and sampling design and attrition (24). This resulted in a final, weighted analytical sample of 17,927 respondents (Diagram S1).

### Measures

#### Frequency of engagement with arts, culture, and heritage

Respondents were asked about, overall, how often they engaged in each type of engagement—namely, arts participation, cultural attendance, and heritage visits—voluntarily in the last 12 months. For arts participation, 14 activities were provided in the list, including dancing, singing, playing an instrument, reading for pleasure, photography, digital arts, and painting and crafting. For cultural attendance, 14 events were included, such as art exhibitions, street art displays, musical concerts, opera, and ballet. For heritage visits, eight historical sites were included, such as visiting a historical park or garden and a monument such as a castle. A full list of the specific activities covered is presented in Table S1. For each type of engagement, the respondents were asked to rate their overall engagement frequency in the listed activities, using a 7-point Likert-type scale ranging from 0 (none in the past 12 months) to 6 (at least once a week).

#### Mental health

Two measures of mental health were analyzed: (i) mental distress and (ii) mental well-being. For mental distress, we used the 12-item GHQ mental distress scale, which measures common symptoms of anxiety and depressive disorder (*α* = 0.91) (25). All items were first summed and then averaged by the number of items, yielding a scale ranging from 1 to 4; higher scores indicate more severe mental distress. For mental well-being, we used the SF-12 Mental Component Summary (MCS) (26), which includes six items relating to vitality, social functioning, role limitations caused by emotional problems, and mental health (*α* = 0.91 at time 1; *α* = 0.90 at time 2). We summed up the scores for all items and averaged the sum by the number of items to create a 1–5 scale, where higher scores indicate better mental well-being.

#### Covariates

Ten covariates that may confound associations between arts and cultural engagement and mental health were included (19, 22, 27). All covariates were measured at time 1 to retain a larger sample size. These included age (years; top-coded at the 99th percentile to minimize the influence of outlier cases) and its quadratic term, gender (men versus women), ethnicity (White ethnic versus ethnic minorities), partnership status (without a cohabiting partner/spouse versus married or cohabiting), living with children aged 15 or under (yes versus no), educational qualification (undergraduate degree or above versus no degree), employment status (in employment versus not in employment), gross household income (British pounds; top-coded at the 99th percentile), living area (rural versus urban), and long-standing mental/physical illness (yes versus no).

We also identified and used a set of IVs to account for unobserved endogeneity and potential reverse causality between arts engagement and mental health, which are discussed in the next section.

### Statistical analysis

To explore the direction of relationships between arts engagement and mental health and strengthen causal inference, we used nonrecursive IV models (28). Compared with a recursive model where all causal effects are unidirectional and error terms are uncorrelated, a nonrecursive IV model allows us to investigate possible concurrent (rather than lagged) bidirectional reciprocal relationships between the arts and mental health, where “feedback loops” between two variables can be tested and error terms are allowed to be correlated. The bidirectional relationships can be tested by jointly estimating multiple equations with multiple dependent variables simultaneously and allowing residuals to be correlated (to obtain joint SEs). Given that nonrecursive models can often be unidentified, IVs were therefore introduced to aid model identification (28, 29).

In our models, we estimated reciprocal paths between arts engagement and mental health at time 2 (i.e. the current time point), respectively, for each type of engagement and each mental health outcome, while accounting for the error terms for both engagement and mental health and their covariance. The models specified IVs as predictors for arts engagement and mental health to account for unobserved endogeneity and potential reverse causality. Two types of IVs were included: auxiliary IVs (AIVs) and model-implied IVs (MIIVs). On the one hand, AIVs are a common type of IV and often include time-lagged variables (30). They can be particularly helpful in identifying the feedback relationship between arts engagement and mental health as they are strongly correlated with the endogenous predictor being instrumented. MIIVs, on the other hand, are identified based on existing knowledge to build a model where particular variables correlate with the predictor being instrumented and are uncorrelated with the error of the equation (30). Combining these two types of IVs, therefore, helped satisfy the four main requirements of IVs: (i) *relevance*—to be strongly correlated with the predictor being instrumented; (ii) *exclusion*—to only affect the outcome of interest through the predictor; (iii) *exchangeability*—to not share common causes with the outcome; and (iv) *effect homogeneity*—the effect of IV on the outcome is constant and homogenous across individuals (31).

Three IVs instrumenting arts engagement were used: (i) arts engagement measured at time 1, i.e. measures of arts participation, cultural attendance, and heritage visits at time 1, respectively; (ii) average regional rate of arts engagement (individually for each type of engagement) at time 1; and (iii) average regional rate of public library visits at time 1. The region measure distinguishes nine broad regions in England, as well as Scotland, Wales, and Northern Ireland. The latter two variables were identified as MIIVs and were expected to have an impact on arts engagement. For example, a study used the average regional cultural attendance rate (alongside book ownership and financial hardship on cinema, theater, opera, or other concert attendance), as IVs instrumenting cultural participation, to estimate the causal relationship between cultural participation and self-reported health and depression (32). Studies on libraries suggested that such spaces can promote arts engagement through enabling library visitors to consume knowledge and acquire new skills (such as on arts and crafts or information on cultural performance or heritage sites) and offering spaces for workshops, seminars, and programs (such as poetry workshops or book clubs) (33). Given that MIIVs were measured on a regional level, they were expected to be strongly associated with and relevant to individual arts engagement and to be related to mental health only through their effects on individual arts engagement (Diagram S2a; Fig. S1 for kernel density estimates of arts engagement by IVs).

Three IVs instrumenting mental health were used: (i) mental health measured at time 1, i.e. measures of mental distress and mental well-being at time 1, respectively; (ii) whether one was diagnosed with clinical depression at time 1; and (iii) whether someone they knew passed away in the past year at time 1. Again, the latter two variables were identified as MIIVs and were expected to have an impact on mental health. A large volume of psychological literature has shown that stressful life events, such as the death of a social contact, are associated with increased major depressive episodes, panic disorder, anxiety, posttraumatic stress disorder, generalized anxiety disorder (34), suicidal ideation, and loneliness (34–37). Although there may be a situation where the person who passed away might have provided resources and opportunities for one to engage in the arts and hence affect their engagement, the immediate shock of knowing someone who passed away is likely to first impact their mental health and well-being and later their behaviors (38). Similarly, clinically diagnosed depression has also been shown to have a direct impact on symptoms of mental distress and well-being (39–41); although diagnosed depression can impact arts engagement, it is usually through the most present state of mental distress and well-being (Diagram S2b; Figs. S2 and S3 for kernel density estimates of mental health by IVs).

To ensure the adequacy of the IVs (42), we used two-stage least squares regression to perform three statistical tests: (i) Kleibergen-Paap rk Langurange Multiplier (LM) statistics, (ii) Kleibergen-Paap rk Wald *F* statistics, and (iii) the Sargan–Hansen test. The null hypothesis of the Kleibergen-Paap rk LM is that the equation is under-identified, testing whether the excluded instruments are uncorrelated with the predictor being instrumented; in other words, whether the instruments are relevant. The Kleibergen-Paap rk Wald *F* statistics measure weak instruments, which occur when their correlation with the predictor being instrumented is low. The Sargan–Hansen test examines overidentifying restrictions, with the null hypothesis that the IVs are valid instruments (i.e. IVs are uncorrelated with the error term and that they are correctly excluded from the estimated equation). These tests showed that, across all models, we can reject the null hypothesis of tests that the equation was under-identified (Kleibergen-Paap rk LM *P* < 0.05) or weakly identified (Wald *F* statistic ranges from 633.4 to 2,130.3). That means the identified IVs were strongly correlated with the predictor being instrumented. The Sargan–Hansen test for all models indicated that the IVs were valid instruments (Sargan–Hansen *P* > 0.05; except for the model where mental well-being was instrumented to estimate its relationship with arts participation where *P* = 0.044). Full tables of these tests for all models are presented in Supplementary material (Table S2 for mental health being instrumented to estimate its relationship with arts engagement; and Table S3 for arts engagement being instrumented to estimate its relationship with mental health).

Once the IVs were identified, we estimated the reciprocal relationships between arts engagement and mental health individually for each type of engagement and measure of mental health, resulting in a total of six nonrecursive IV models. All variables were standardized to aid the interpretation of results. The models included endogenous predictors measured at time 2 to test for concurrent reciprocity. AIVs, MIIVs, and a set of covariates measured at time 1 were also included.

One advantage of nonrecursive models is that they allow for assessing the concurrent bidirectional relationships between arts engagement and mental health in the same wave simultaneously. Yet, it is also possible that previous arts engagement may also influence current mental health, and previous mental health may influence current arts engagement. To test such lagged bidirectionality, we ran cross-lagged structural equation models as an additional check to explore a potential temporal ordering between arts engagement and mental health. Results from cross-lagged models are likely to be more conservative, since there was a time gap of ∼3 years between time 1 and time 2. Respondents’ current arts engagement is likely to be a more relevant predictor of their current mental health than their engagement 3 years ago (and vice versa) (43). Despite this, if results from both nonrecursive and cross-lagged structural equation models were consistent, this would provide stronger evidence for a causal relationship between arts engagement and mental health, which has been the case in our findings.

For both nonrecursive IV models and cross-lagged structural equation models, standardized root mean squared residual (SRMR) and coefficient of determination (CD) were estimated to evaluate model fit. The models showed SRMR <0.05 and a CD between 0.426 and 0.600, indicating the models were well fit (44). All analyses were performed using Stata version 18.0.

## Results

### Participant characteristics

The average age of the sample was 47.8 years (SD = 14.3) at time 1, 52.7% were female, 92.5% were of White ethnicity, 24.6% had a degree or higher qualification, and 59.5% were employed. Around 76.8% of the sample lived in an urban area and 35.2% had a long-standing mental/physical illness. More than half of the sample (55.8%) engaged with arts at least once a week, 18.1% attended cultural events, and 12.3% visited heritage sites at least once a month. On average, the sample had a mean score of 1.92 (SD = 0.36, range: 1–4) on the GHQ-12 (measuring mental distress) and 4.04 (SD = 0.58, range: 1–5) on the SF-12 MCS (measuring mental well-being) at time 1 (Table S4 and Fig. S4 show levels of arts engagement at times 1 and 2).

### Concurrent directionality

**Fig. 1.**
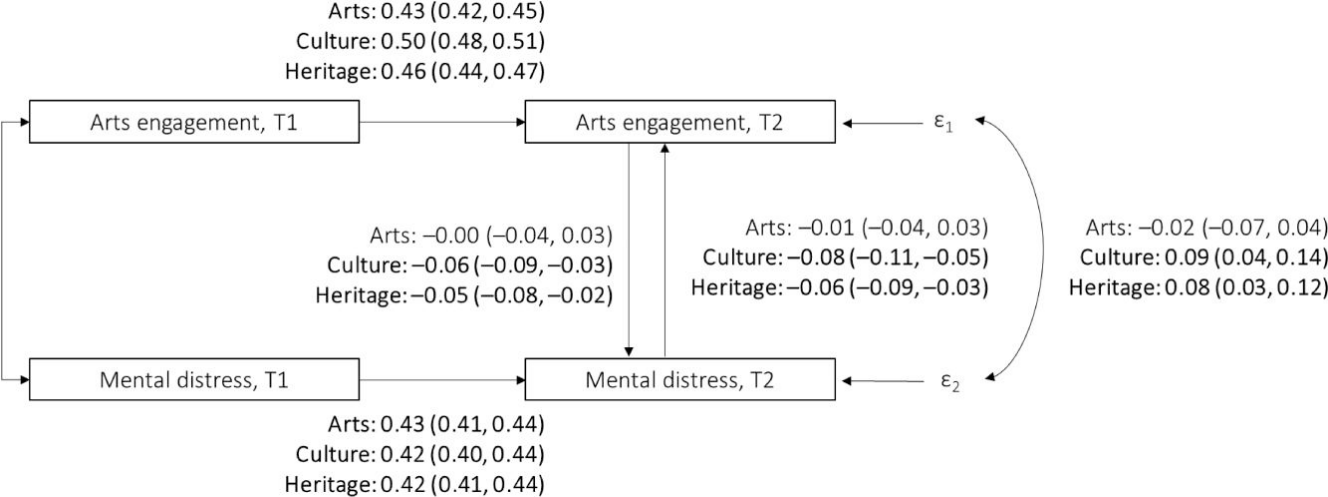
Nonrecursive IV structural equation models estimating the bidirectional relationships between engagement with arts, culture and heritage, and mental distress (modeled separately for each type of engagement). All models controlled for all covariates are listed in Table S4. Three IVs instrumenting arts engagement were included: arts engagement measured at time 1, average regional rates of arts engagement, and average regional rate of public library visits. Three IVs instrumenting mental distress were included: mental distress measured at time 1, diagnosed clinical depression, and knowing someone who passed away in the past year. T1 = time 1, wave 2. T2 = time 2, wave 5. Standardized beta and 95% CI (in parentheses) are presented. Bold values denote statistical significance at the *P* < 0.05 level, with specific *P-*values presented in the main text.

As shown in Fig. 1, a reciprocal relationship was observed at time 2 between cultural attendance and heritage visits and mental distress, such that more frequent engagement was associated with lower mental distress (also see Tables S5a–S5c). Specifically, every one-SD increase in cultural engagement reduced mental distress by 0.06 SD (95% CI = −0.09 to −0.03; *P* < 0.001). For heritage visits, every one-SD increase in visits reduced mental distress by 0.05 SD (95% CI = −0.08 to −0.02; *P* = 0.004). Simultaneously, higher mental distress was associated with less frequent cultural attendance and heritage visits, with every one-SD increase in mental distress reducing cultural attendance by 0.08 SD (95% CI = −0.11 to −0.05; *P* < 0.001) and heritage visits by 0.06 SD (95% CI = −0.09 to −0.03; *P* < 0.001). No associations were found between arts participation and mental distress.

**Fig. 2.**
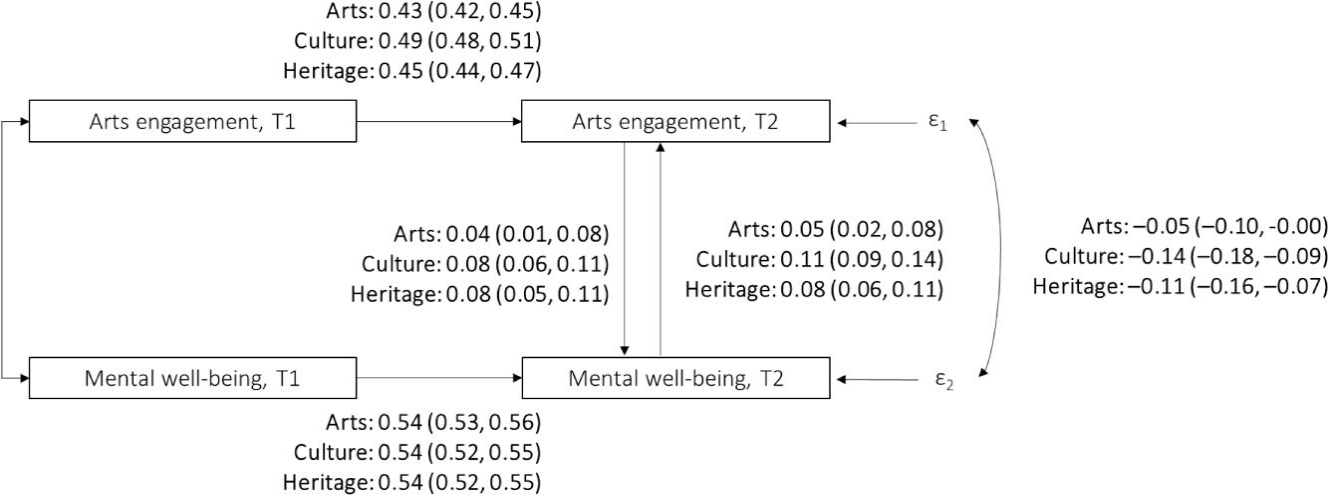
Nonrecursive IV structural equation models estimating the relationships between engagement with arts, culture and heritage, and mental well-being (modeled separately for each type of engagement). All models controlled for all covariates are listed in Table S4. Three IVs instrumenting arts engagement were included: arts engagement measured at time 1, average regional rates of arts engagement, and average regional rate of public library visits. Three IVs instrumenting mental well-being were included: mental well-being measured at time 1, diagnosed clinical depression, and knowing someone who passed away in the past year. T1 = time 1, wave 2. T2 = time 2, wave 5. Standardized beta and 95% CI (in parentheses) are presented. Bold values denote statistical significance at the *P* < 0.05 level, with specific *P-*values presented in the main text.

As Fig. 2 shows, a reciprocal relationship was also found at time 2 for mental well-being and all types of engagement (also see Tables S6a–S6c). Specifically, every one-SD increase in arts participation, cultural engagement, and heritage visits increased mental well-being by 0.04 SD (95% CI = 0.01 to 0.08; *P* = 0.009), 0.08 SD (95% CI = 0.06 to 0.11; *P* < 0.001), and 0.08 SD (95% CI = 0.05 to 0.11; *P* < 0.001), respectively. Simultaneously, better mental well-being was associated with higher levels of arts participation (β = 0.05, 95% CI = 0.02 to 0.08; *P* = 0.002), cultural attendance (β = 0.11, 95% CI = 0.09 to 0.14; *P* < 0.001), and heritage visits (β = 0.08, 95% CI = 0.06 to 0.11, *P* < 0.001).

### Lagged directionality

**Fig. 3.**
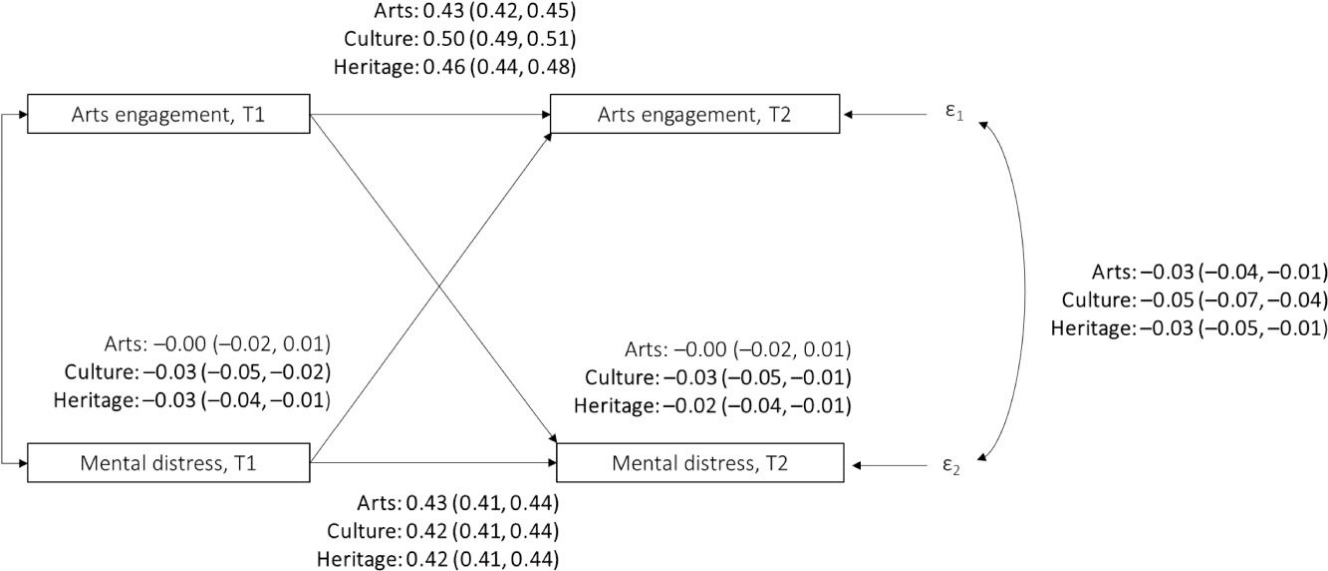
Cross-lagged structural equation models estimating the relationships between engagement with arts, culture and heritage, and mental distress (modeled separately for each type of engagement). All models controlled for all covariates are listed in Table S4 and adjusted for time 1 arts engagement (in the equation predicting time 2 arts engagement) or mental health (in the equation predicting time 2 mental health). Two IVs instrumenting arts engagement were included: average regional rates of arts engagement and average regional rate of public library visits. Two IVs instrumenting mental well-being were included: diagnosed clinical depression and knowing someone who passed away in the past year. T1 = time 1, wave 2. T2 = time 2, wave 5. Standardized beta and 95% CI (in parentheses) are presented. Bold values denote statistical significance at the *P* < 0.05 level, with specific *P-*values presented in the main text.

When considering how arts engagement at time 1 may also influence mental health at time 2 and vice versa, we found that a one-SD increase in mental distress at time 1 was associated with a reduction in cultural attendance by 0.03 SD (95% CI = −0.05 to −0.02; *P* < 0.001) and heritage visits by 0.03 SD (95% CI = −0.04 to −0.01, *P* < 0.001) 3 years later (Fig. 3; Tables S7a–S7c). People who attended cultural events (β = −0.03, 95% CI = −0.05 to −0.01; *P* < 0.001) or heritage sites (β = −0.02, 95% CI = −0.04 to −0.01; *P* = 0.004) less often at time 1 experienced greater mental distress at time 2. Concurring with the results from the nonrecursive models discussed in the preceding section, no associations were found between arts participation at time 1 and mental distress at time 2.

**Fig. 4.**
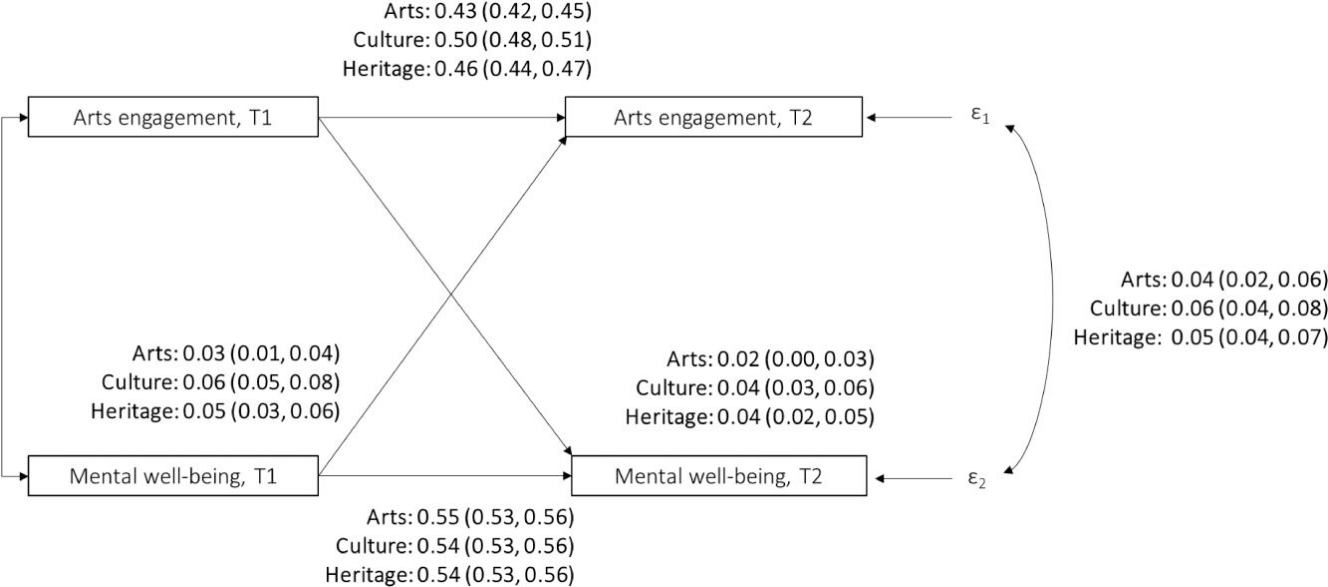
Cross-lagged structural equation models estimating the relationships between engagement with arts, culture and heritage and mental well-being (modeled separately for each type of engagement). All models controlled for all covariates are listed in Table S4 and adjusted for time 1 arts engagement (in the equation predicting time 2 arts engagement) or mental health (in the equation predicting time 2 mental health). Two IVs instrumenting arts engagement were included: average regional rates of arts engagement and average regional rate of public library visits. Two IVs instrumenting mental well-being were included: diagnosed clinical depression and knowing someone who passed away in the past year. T1 = time 1, wave 2. T2 = time 2, wave 5. Standardized beta and 95% CI (in parentheses) are presented. Bold values denote statistical significance at the *P* < 0.05 level, with specific *P-*values presented in the main text.

For mental well-being, Fig. 4 shows that those with better mental well-being at time 1 participated in arts activities (β = 0.03, 95% CI = 0.01 to 0.04; *P* = 0.002), attended cultural events (β = 0.06, 95% CI = 0.05 to 0.08; *P* < 0.001), and visited heritage sites (β = 0.05, 95% CI = 0.03 to 0.06; *P* < 0.001) more frequently at time 2 (also see Tables S8a–S8c). People who engaged more in these activities at time 1 were also more likely to report better mental well-being 3 years later (arts participation: β = 0.02, 95% CI = 0.00 to 0.03; *P* = 0.010; cultural attendance: β = 0.04, 95% CI = 0.03 to 0.06; *P* < 0.001; heritage visits: *β* = 0.04, 95% CI = 0.02 to 0.05; *P* < 0.001).

## Discussion

This study addressed important theoretical and methodological questions surrounding the directionality of the relationship between engagement with the arts, culture and heritage, and mental health, and found that this relationship to be causally bidirectional. After adjusting for time 1 measures and identifying confounders, cultural attendance and heritage visits are reciprocally associated with mental distress and mental well-being, while arts participation is only reciprocally associated with mental well-being. The bidirectional causal effects between arts engagement and mental health are relatively modest in scale, which may be anticipated given arts are one aspect of people's broader characteristics and environment. But these effects are clearly present not only in mental health to arts, but also in arts to mental health. Cross-lagged models examining lagged directionality with a 3-year interval supported our main findings of bidirectional effects, showing that previous engagement with arts, culture, and heritage affects current mental health and prior mental health affects current engagement.

Previous studies suggested that mental health could be both predictor and result of arts engagement, but mostly in two separate bodies of literature (3, 14–16), implying reciprocal effects between arts engagement and mental health. Very few studies have systematically investigated the potentially reciprocal relationship between arts engagement and mental health, leaving the confirmation of bidirectionality and the relative magnitude of both directions of effect unclear. Using nonrecursive models to parse out these effects, our analyses show that arts engagement is linked with a lower level of mental distress and a higher level of mental well-being, and people with better mental health also engage in participatory arts activities and attend cultural events and heritage sites more frequently. Importantly, the reciprocal effects remain after controlling for demographic background, socioeconomic position, and health profile. The use of IVs, including previous levels of arts engagement/mental health and variables commonly used as IVs (such as average regional engagement rates in arts, culture, and heritage), has further strengthened the ability of our models to identify concurrent (rather than lagged) reciprocal effects. Our IV nonrecursive approach indicates that the bidirectional relationships between arts engagement and mental health are not just a result of related economic, cultural, and social capital. Instead, arts engagement can itself confer mental health benefits, and mental health can influence whether one engages in the arts. This finding is consistent with a Swedish study on a working population that used a multilevel structural equation modeling approach and found a lagged bidirectional association between cultural activity provided at the workplace and depressive symptoms among employees (45). Our use of an IV approach to identify concurrent bidirectional relationships across engagement types and multidimensional measures of mental health further strengthens the evidence for the bidirectional association (45). Our results invite careful critical reflections on the potential “vicious” and “virtuous” cycles between arts engagement and mental health.

A vicious cycle occurs when low arts engagement and poor mental health reinforce each other, in which the situation may spiral in a downward loop and worsen over time. There are several theoretical reasons why individuals with poorer mental health may experience more barriers to engaging in the arts. These barriers are often related to their capabilities, opportunities, and motivations (46). For instance, one study found that people with depression and anxiety were more likely to report feeling less psychologically and physically capable of engaging (e.g. limited awareness of different types of activities available) and report having fewer social opportunities (16). They were also more likely to report feeling lower enjoyment and fewer perceived benefits from engaging in the arts (16). These barriers can be persistent, leaving people of poorer mental health with fewer coping options to improve their health. The impacts of mental health on arts engagement could also be understood through the expectancy-value theory (47). This theory suggests that arts engagement decisions can be influenced by two sets of beliefs: expectations of success (how one perceives their abilities in arts and culture) and subjective task value (how one perceives enjoyment, usefulness, importance, and cost of engagement) (47). People with poorer mental health may hold these beliefs more negatively and may thus feel less motivated to engage in the arts. Over time, this vicious cycle can become very difficult to break without extrinsic intervention.

On the flip side, a virtuous cycle occurs when a person who engages in the arts has better mental health, which provides greater incentives for them to stay engaged with the activities and then continues to support their mental health. As proposed in the Health Belief Model (48), people may be more likely to engage in an activity if they perceive benefits in such engagement. Applying this lens, individuals who recognize the health benefits of the arts may be more likely to engage in the activities, and such recognition may be more commonly found in individuals with better mental health. Furthermore, arts engagement often involves a “social” active ingredient, in which the social elements of arts engagement, through either involving social contacts or facilitating social interactions, could have an additional impact in improving people's mental health through developing social capital and group belonging (3). An increase in social capital and group belonging may keep people in the loop of engagement and promote future engagement, as highlighted by the social identity theory (49).

Consequently, the vicious and virtuous cycles may widen health inequalities between people with better and poorer mental health, and between those who engaged and disengaged with the arts. This suggests that the arts system and public health system may mutually reinforce one another in constituting social and health stratification, and intervention approaches that increase people's engagement in the arts or that mitigate poor mental health could help prevent any negative feedback that may occur between the two. While interventions in either arts engagement or mental health would likely also alter the other, encouraging arts engagement may be a more feasible approach to breaking the negative feedback loop.

Another notable finding of our study is that, unlike cultural attendance and heritage visits, arts participation is only reciprocally associated with mental well-being but not mental distress. One potential explanation may be that arts participation, which often involves active and creative engagement, may be more strongly associated with components of mental well-being (such as vitality, social well-being, and emotional well-being). It is plausible that such engagement may play a bigger role in improving one's positive emotions than reducing negative emotions, such as mental distress, anxiety, and depression. Conversely, people with a higher level of mental distress may be more attracted to receptive activities, such as attending cultural events and visiting heritage sites to boost their mental health, which require less effort. In parallel studies that focused on the frequency of using general practitioner (GP) consultations (50) and cause-specific mortality (51), differences were also observed in the effects of active and creative engagement versus receptive engagement. This highlights that different types of engagement involving various key active ingredients (9) and mechanisms of action (10) may have differential health impacts. It is, therefore, crucial to provide a variety of activities for people with different mental health needs to access and participate. Alternatively, given that our definition of arts participation included activities that could be performed alone, individuals experiencing mental distress may have felt more motivational barriers to solo engagement, but may have been more likely to engage in culture and heritage activities that are frequently done with others or involve passing informal social contact as part of engaging, thereby providing both the social pressure to engage in the first place and social confirmation in support of that engagement (9). The lack of association between arts participation and mental distress may also be related to artistic abilities that are more relevant to arts engagement (such as playing a musical instrument, painting, and photography).

Our results help inform the theoretical and practical development of interventions and policies across arts and culture, mental health care, and public health sectors. In mental health-care settings, there has been an increasing emphasis on integrating psychological, social, biomedical, and behavioral approaches to improving mental health (52) and supporting the health-care system and mental health professionals. Offering arts, cultural, and heritage activities within mental health-care settings can provide alternatives or complements to pharmacological prescribing, potentially reducing pharmacological prescriptions and the associated side-effects (53, 54). In the context of public health, it has been shown that one in five appointments in general practice in the United Kingdom are for social reasons, such as loneliness (55), which are linked to socioeconomic inequalities such as unemployment, poverty, and discrimination. As socioeconomic position, lifestyle, and culture play a substantial role in people's mental health (56), mental health conditions may be better managed with the additional inclusion of a creative-based approach that involves engagement in arts, cultural, and heritage activities (57). Creative-based approaches are sustainable and scalable, presenting a potential strategy with less-stigma attached than medical approaches to meet the pressure from the growing population with mental ill health. For instance, a recent economic analysis found that a social prescribing intervention led to 4.7 fewer GP appointments and a direct cost saving of £78.4 per participant over a 5-month period (58). In addition, policies expanding access to the arts, especially for individuals with poor mental health, are important in breaking any negative feedback loops. Overall, the positive implications of arts engagement on mental health provide further support for social prescribing and other related creative-based mental health interventions that help improve population mental health.

Our study has many strength, including demonstrating both concurrent and lagged reciprocal relationships between arts engagement and mental health, as well as comparing the magnitude of these relationships. The rich UKHLS data enabled us to use sophisticated and robust statistical models (both nonrecursive models and cross-lagged models) with unique IVs to make stronger conclusions about whether the relationships are causal. We were also able to comprehensively test different types of activities including arts participation, cultural attendance, and heritage visits. However, the study is not without limitations. First, while the GHQ-12 and SF12 are widely used for screening common mental disorders and for assessing the impact of health on everyday life, it remains to be explored whether the reciprocity found in this study would be replicated if they were to be replaced by other mental health measures, such as depression and anxiety. Second, the question remains as to whether the strength of the reciprocal relationships identified in our study is static or evolve across the life course and how long the relationships last. Although we adjusted for a range of potential confounders and utilized the IV approach, unmeasured heterogeneities could still have influenced our findings. Third, respondents were asked about their overall frequency of arts engagement (regardless of whether they had engaged in one or more than one activity). This means their engagement frequency could be influenced by the number of activities in which they were engaged. Relatedly, since measures of arts engagement were only captured in waves 2 and 5 in UKHLS (2010–2015), our study was not able to include more recent engagement forms such as creating videos on social media. Despite this, UKHLS contains a wealth of variables that could be used as covariates and IVs, enabling us to investigate the bidirectional relationship between arts engagement and mental health in a representative sample of the UK population. Building on our baseline study and findings, new data collection will enable researchers to examine change and continuity in the bidirectional relationships between the arts and mental health.

This study has addressed a key theoretical question regarding the direction of causality that is often raised in arts and health research. It suggests that interventions that increase people's engagement in the arts or that mitigate poor mental health may help prevent or break any negative feedback loops between arts engagement and mental health. Future research should consider the potential bidirectional relationships when investigating the antecedents and/or consequences of arts engagement. The findings have several implications for practice. For people with poorer mental health, enhancing routine participation in arts and cultural activities may help to improve their mental health by reducing mental distress and improving mental well-being. For the health sector, building a strong partnership with the arts and cultural sector may help formulate more personalized, nonpharmacological treatments. Finally, if people with poorer mental health engage less in arts and cultural activities, exploring ways to encourage their engagement and removing barriers and obstacles is key. These could involve co-designing a treatment plan with a health-care professional or making the arts more accessible by providing various delivery formats. Given increasing policy attention to the arts and health link from international bodies like the World Health Organization, more evidence on the causal and reciprocal associations between the arts and mental health will allow us to have a better understanding of the potential mental health benefits of arts engagement and how to effectively encourage arts engagement to promote public health. While our analysis establishes new evidence of the bidirectional associations between the arts and mental health averaged across the population, future research could build on our study to further examine sociodemographic heterogeneities in such links to help develop more targeted interventions.

## Supplementary Material

pgae465_Supplementary_Data

## Data Availability

Understanding Society—The UKHLS data can be retrieved via the UK Data Service: https://beta.ukdataservice.ac.uk/datacatalogue/series/series?id=2000053. Data documentation is available from the Understanding Society website: https://www.understandingsociety.ac.uk/documentation. All codes used for the main and supplementary analyses are publicly available via the Open Science Framework: https://osf.io/ar8s7/.
