# Supplementary material for: Art for health’s sake or health for art’s sake: Disentangling the bidirectional relationships between arts engagement and mental health
Source: PNAS Nexus. 2024 Oct 17;3(11):pgae465. doi: 10.1093/pnasnexus/pgae465 (PMC11586668; doi:10.1093/pnasnexus/pgae465)
Supplement: pgae465_Supplementary_Data [file pgae465_supplementary_data.pdf]

Supplementary Materials

for

Art for health's sake or health for art's sake: Disentangling the bidirectional relationships between arts engagement and mental health

Contents

Diagram S1: A flowchart of analytic sample construction 2

Diagrams S2a & b: Path diagrams for instrumental variables 3

Figures S1-S3 Density estimates of arts engagement and mental health by instrumental variables 4

Figure S4: Level for arts engagement at Times 1 & 2 6

Table S1: The listed activities for arts participation, cultural attendance and heritage visits 7

Tables S2 & S3: Two-stage least squares (2SLS) regression 8

Table S4: Sample characteristics from weighted and unweighted data 10

Tables S5a-S6c: Non-recursive instrumental–variable models 11

Tables S7a-S8c: Cross–lagged models 17

Diagram S1: A flowchart of analytic sample construction

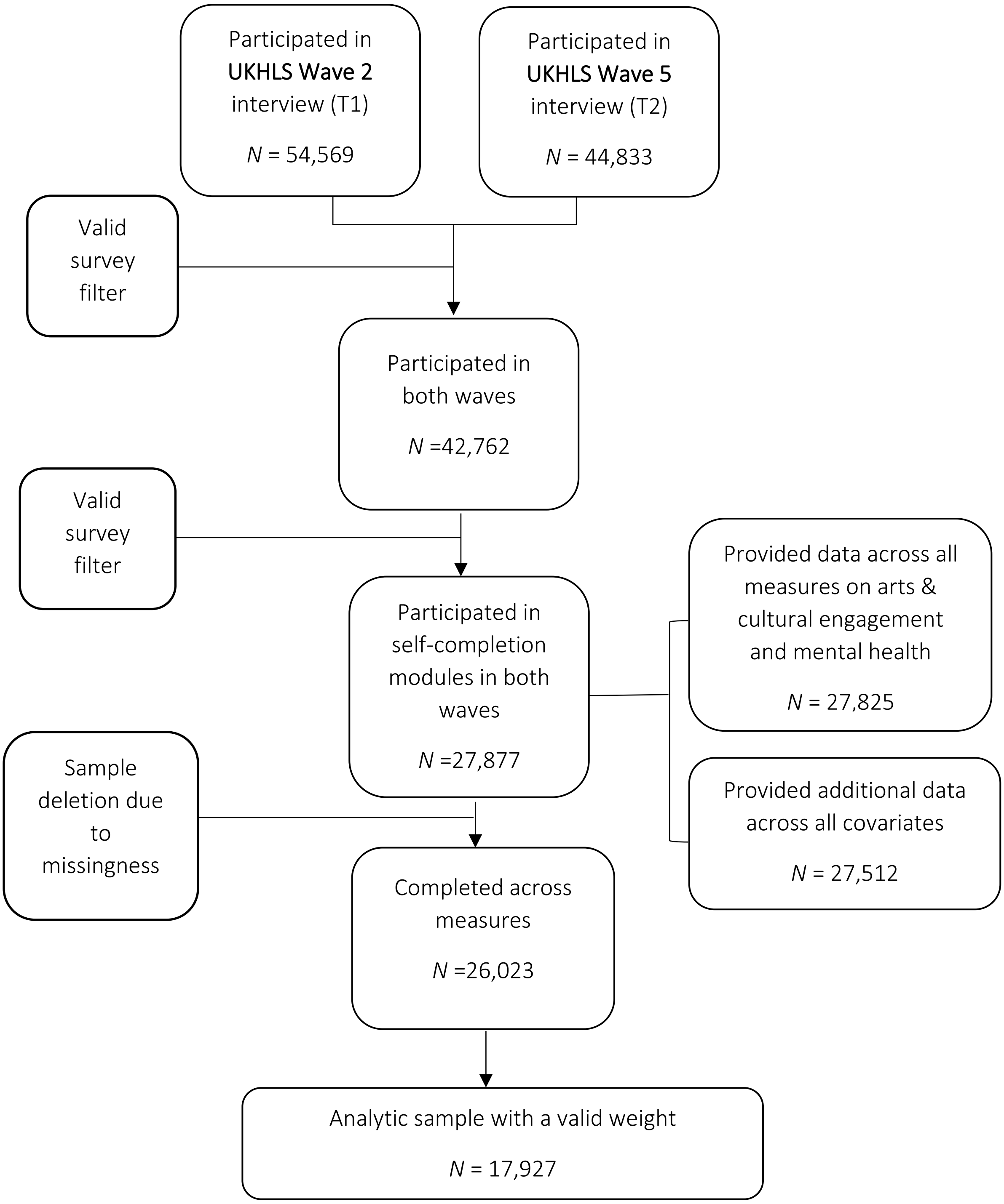

UKHLS = Understanding Society: The UK Household Longitudinal Study. T1 = Time 1 (Wave 2). T2 = Time 2 (Wave 5).

Diagrams S2a & b: Path diagrams for instrumental variables

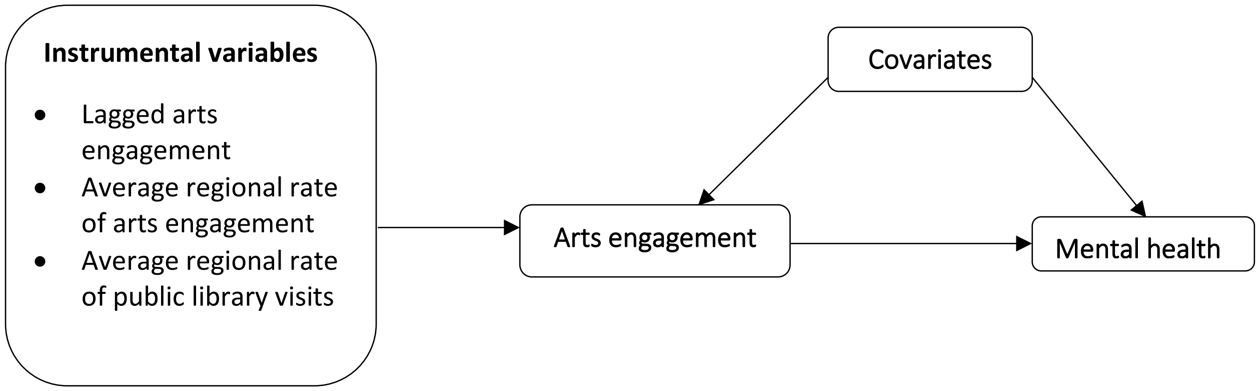

Diagram S2a: Instrumental variables instrumenting arts engagement.

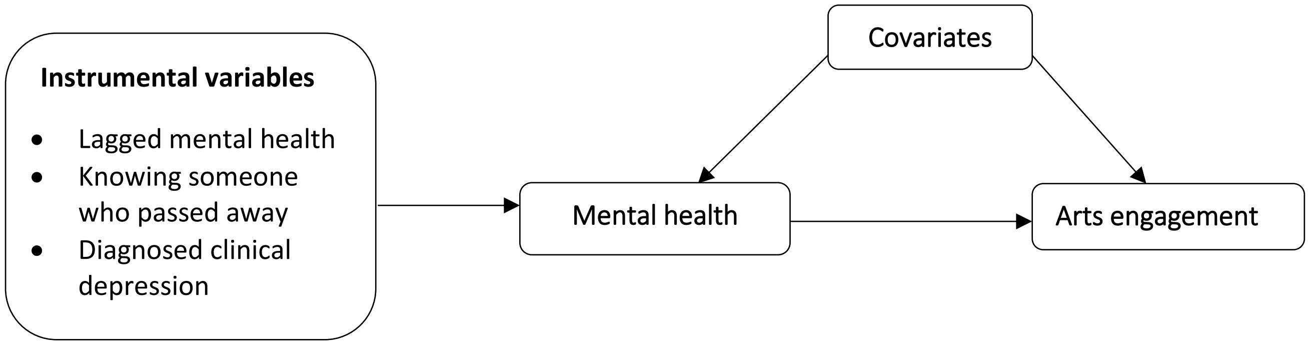

Diagram S2b: Instrumental variables instrumenting mental health.

Figures S1-S3 Density estimates of arts engagement and mental health by instrumental variables

Figure S1: Density estimates of arts engagement by average regional engagement level

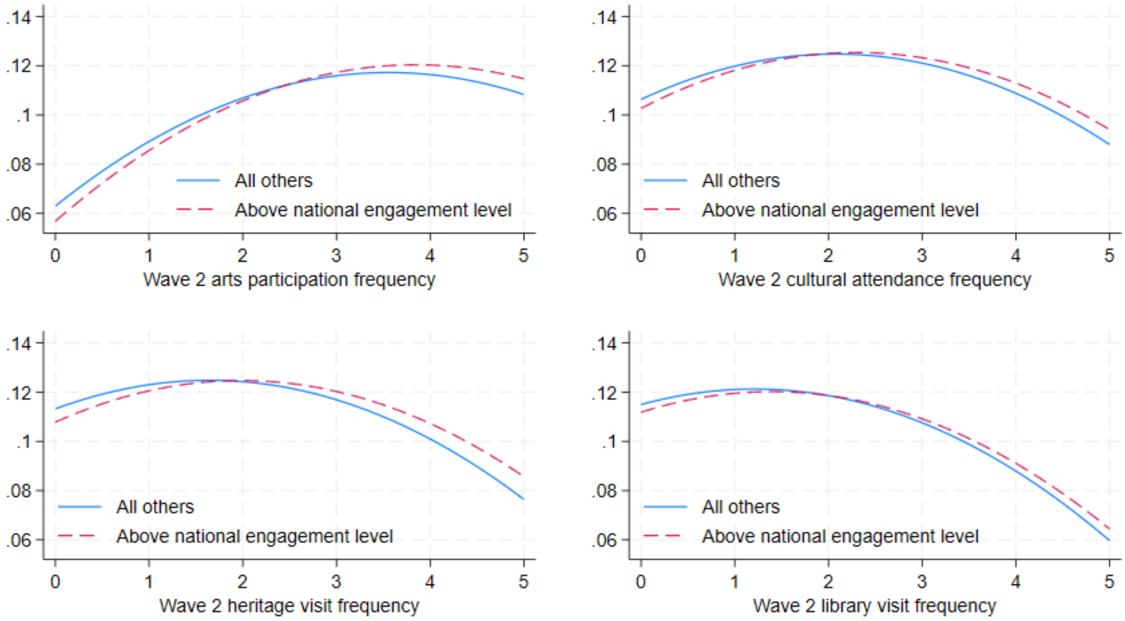

Figure S2: Density estimates of mental mealth by knowing someone died last year

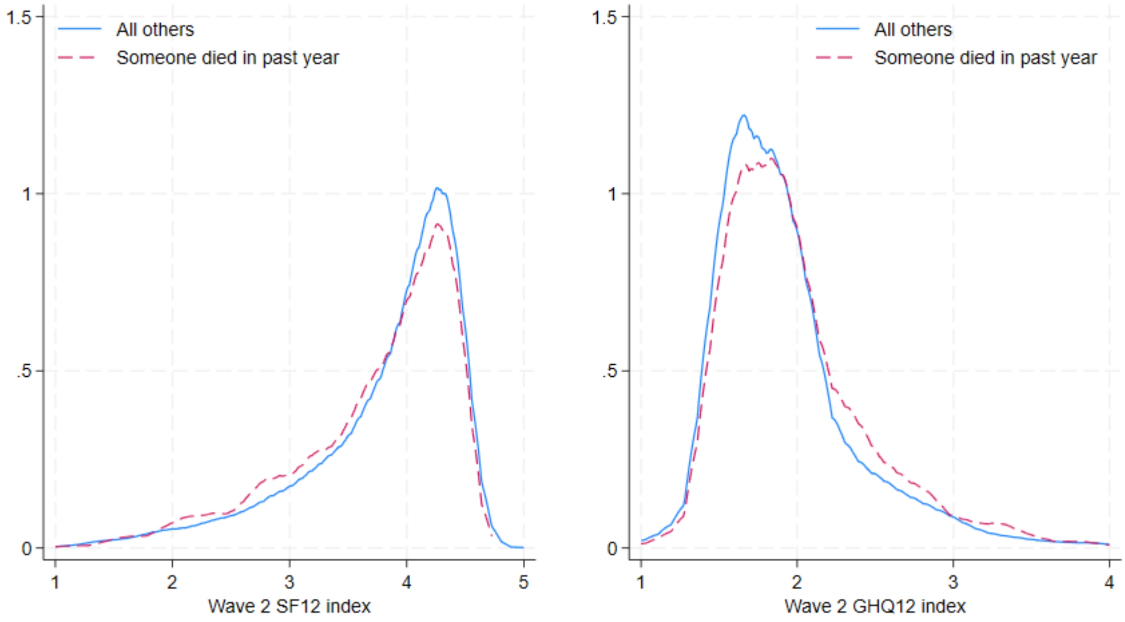

Figure S3: Density estimates of mental health by depression diagnosis

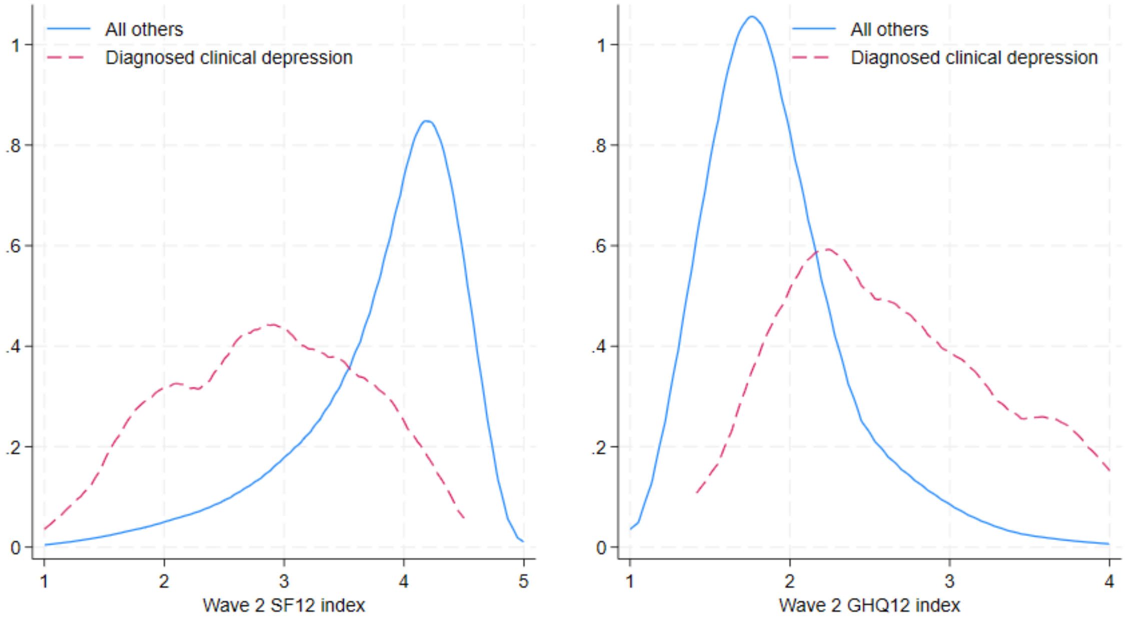

Figure S4: Level for arts engagement at Times 1 & 2

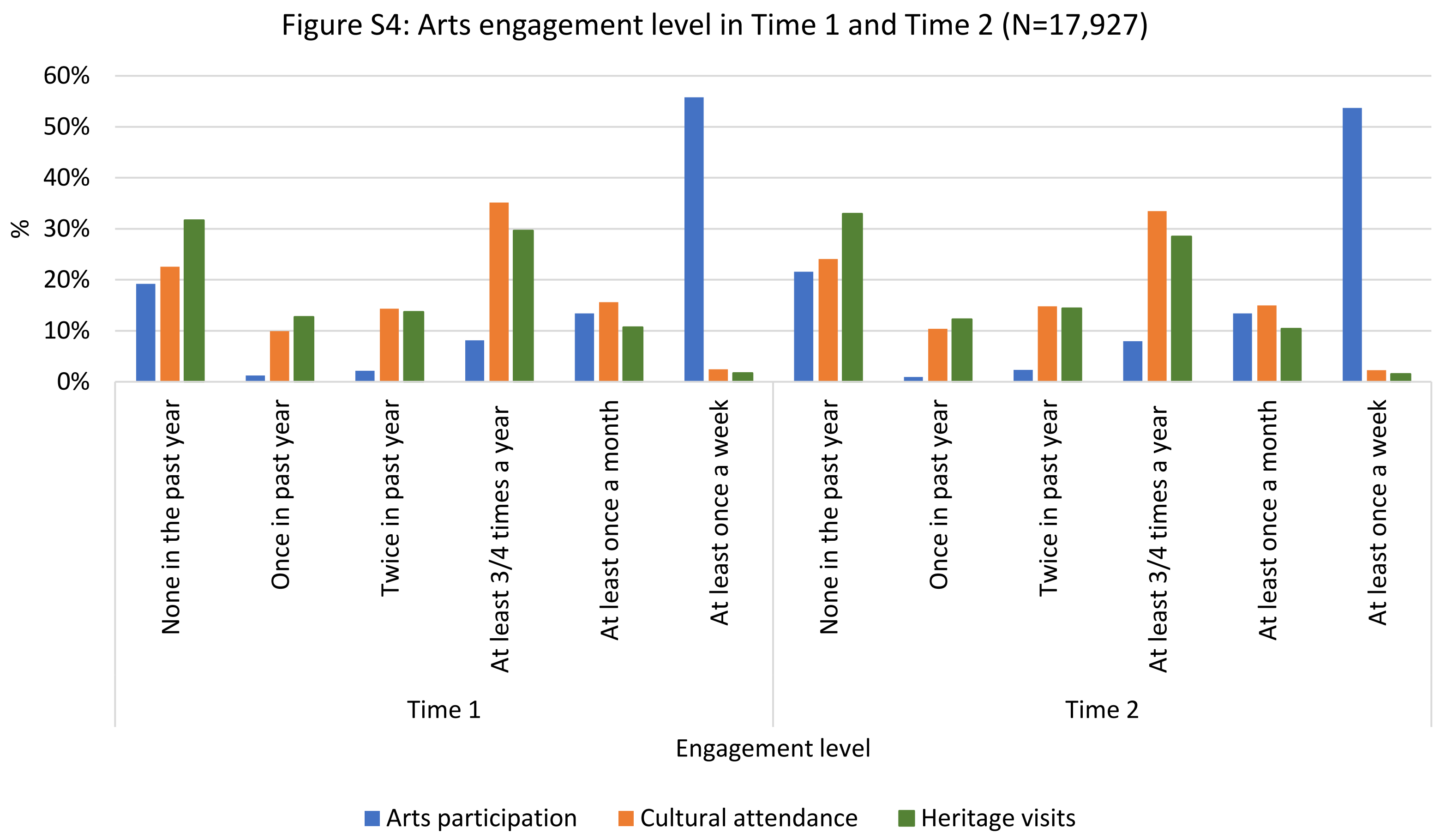

Table S1: The listed activities for arts participation, cultural attendance and heritage visits

Arts participation
Dance, including ballet<br>Sang to an audience or rehearsed for a performance (not karaoke)<br>Played a musical instrument<br>Written music<br>Rehearsed or performed in play/drama, opera/operetta or musical theatre<br>Taken part in carnival or street arts event (e.g. as a musician, dancer or costume maker)<br>Learned or practised circus skills<br>Painting, drawing, printmaking or sculpture<br>Photography, film or video making as an artistic activity (not family or holidays)<br>Used a computer to create original artworks or animation<br>Textile crafts, wood crafts, or any other crafts, such as embroidery, knitting, wood turning, furniture making, pottery or jewellery<br>Read for pleasure (not newspapers, magazines or comics)<br>Written any stories, plays or poetry<br>Been a member of a book club, where people meet up to discuss and share books.
Cultural attendance
Film at a cinema or other venue<br>Exhibition or collection of art, photography or sculpture or a craft exhibition (not crafts market)<br>Event which included video or electronic art<br>Event connected with books or writing<br>Street arts or public art display or installation (arts in everyday surroundings, or an art work such as sculpture that is outdoors or in a public place)<br>Carnival or culturally specific festival (e.g. Mela, Baisakhi, Navrati, Feis)<br>Circus (not animals)<br>Play/drama, pantomime or a musical<br>Opera/operetta<br>Classical music performance<br>Rock, pop or jazz performance<br>Ballet<br>Contemporary dance<br>African people’s dance or South Asian and Chinese dance.
Heritage visits
A city or town with historic character<br>A historic building open to the public (non-religious)<br>A historic park or garden open to the public<br>A place connected with industrial history (e.g. an old factory, dockyard or mine) or historic transport system (e.g. and old ship or railway)<br>A historic place of worship attended as a visitor (not to worship)<br>A monument such as a castle, fort or ruin<br>A site of archaeological interest (e.g. Roman villa, ancient burial site)<br>A site connected with sports heritage (e.g. Wimbledon) (not visited for the purposes of watching sport)

Tables S2 & S3: Two-stage least squares (2SLS) regression

Table S2: Two-stage least squares (2SLS) regression estimating the relationship between mental health (T2) and arts engagement (T2) (*N* = 17,927) - mental health being instrumented

	Arts participation, T2			Cultural attendance, T2			Heritage visits, T2		
	Coef.	95%CI	p-value	Coef.	95%CI	p-value	Coef.	95%CI	p-value
Mental distress, T2	–0.21	–0.38, –0.04	0.017	–0.54	–0.65, –0.42	< 0.001	–0.40	–0.52, –0.29	< 0.001
Kleibergen-Paap rk LM statistic	1184.014		< 0.001	1184.014		< 0.001	1184.014		< 0.001
Kleibergen-Paap rk Wald F statistic	633.363			633.363			633.363		
Sargan-Hansen $\chi 2$	1.635		0.4416	1.731		0.4209	0.154		0.9257
Mental well-being, T2	0.47	0.39, 0.56	< 0.001	0.57	0.51, 0.63	< 0.001	0.50	0.44, 0.56	< 0.001
Kleibergen-Paap rk LM statistic	2353.746		< 0.001	2353.746		< 0.001	2353.746		< 0.001
Kleibergen-Paap rk Wald F statistic	2130.260			2130.260			2130.260		
Sargan-Hansen $\chi 2$	6.271		0.0435	3.179		0.2041	1.998		0.3683

Note: T1 = Time 1, Wave 2. T2 = Time 2, Wave 5. All models controlled for age, age-squared, gender, ethnicity, partnership status, living with children aged 15 or under, educational qualification, employment status, gross household income, living area, and long-standing mental/physical illness. Both mental health measures were instrumented by (a) mental distress and mental well-being at Time 1 (individually for each model), (b) diagnosed clinical depression, and (c) knowing someone who passed away in the past year.

Table S3: Two-stage least squares (2SLS) regression estimating the relationship between arts engagement (T2) and mental health (T2) (*N* = 17,927) - arts engagement being instrumented						
	Mental distress, T2			Mental well-being, T2		
	Coef.	95%CI	p-value	Coef.	95%CI	p-value
Arts participation, T2	–0.01	–0.02, –0.00	0.017	0.06	0.05, 0.07	< 0.001
Kleibergen-Paap rk LM statistic	1794.524		< 0.001	1794.524		< 0.001
Kleibergen-Paap rk Wald F statistic	884.867			884.867		
Sargan-Hansen $\chi 2$	0.558		0.7565	3.173		0.2046
Cultural attendance, T2	–0.04	–0.05, –0.03	< 0.001	0.12	0.10, 0.13	< 0.001
Kleibergen-Paap rk LM statistic	2975.274		< 0.001	2975.274		< 0.001
Kleibergen-Paap rk Wald F statistic	1503.317			1503.317		
Sargan-Hansen $\chi 2$	0.704		0.7031	1.165		0.5584
Heritage visits, T2	–0.03	–0.04, –0.02	< 0.001	0.12	0.10, 0.13	< 0.001
Kleibergen-Paap rk LM statistic	2588.229		< 0.001	2588.229		< 0.001
Kleibergen-Paap rk Wald F statistic	1241.250			1241.250		
Sargan-Hansen $\chi 2$	0.402		0.8180	0.458		0.7952
Note: T1 = Time 1, Wave 2. T2 = Time 2, Wave 5. All models controlled for age, age-squared, gender, ethnicity, partnership status, living with children aged 15 or under, educational qualification, employment status, gross household income, living area, and long-standing mental/physical illness. All arts engagement were instrumented by (a) arts participation, cultural attendance and heritage visits at Time 1 (individually for each model), (b) average regional rates of arts participation, cultural attendance and heritage visits (individually for each model), and (c) average regional rate of public library visits.						

Table S4: Sample characteristics from weighted and unweighted data

Table S4: Sample characteristics from weighted and unweighted data

	Weighted (N = 17,927) Mean (SD) or %	Unweighted (N = 26,031) Mean (SD) or %
**Arts engagement frequency, T1**		
Arts participation, ranging from 0 to 5	3.63 (1.60)	3.62 (1.96)
Cultural attendance, ranging from 0 to 5	2.19 (1.21)	2.16 (1.48)
Heritage visits, ranging from 0 to 5	1.80 (1.23)	1.76 (1.49)
**Arts engagement frequency, T2**		
Arts participation, ranging from 0 to 5	3.52 (1.65)	3.52 (2.02)
Cultural attendance, ranging from 0 to 5	2.12 (1.22)	2.09 (1.48)
Heritage visits, ranging from 0 to 5	1.76 (1.23)	1.71 (1.49)
**Mental health, T1**		
Mental distress, ranging from 1 to 4	1.92 (0.36)	1.93 (0.45)
Mental well-being, ranging from 1 to 5	4.04 (0.58)	4.02 (0.72)
**Mental health, T2**		
Mental distress, ranging from 1 to 4	1.92 (0.38)	1.93 (0.46)
Mental well-being, ranging from 1 to 5	3.96 (0.60)	3.95 (0.73)
**Instrumental variables, T1**		
Average regional rate of arts participation, ranges from 3.01 to 3.75	3.37 (0.19)	3.37 (0.22)
Average regional rate of cultural attendance, ranging from 1.93 to 2.29	2.06 (0.10)	2.05 (0.11)
Average regional rate of heritage visits, ranging from 1.21 to 1.90	1.58 (0.18)	1.57 (0.21)
Average regional rate of public library visits, ranging from 1.04 to 1.50	1.20 (0.11)	1.18 (0.13)
Not diagnosed clinical depression	98.4%	98.3%
Diagnosed clinical depression	1.62%	1.66%
No one passed away	91.5%	91.2%
Someone passed away	8.53%	8.85%
**Covariates, T1**		
Age, ranging from 16 to 85 (top-coded at 99th percentile)	47.8 (14.3)	48.7 (16.6)
Male	47.3%	42.4%
Female	52.7%	57.6%
Ethnic minorities	7.50%	9.85%
White ethnic	92.5%	90.2%
Not married or not in cohabitation	34.6%	32.3%
Married or in cohabitation	65.5%	67.7%
Not living with children under the age of 15 or below	68.8%	67.2%
Living with children under the age of 15 or below	31.2%	32.9%
Without degree	75.4%	76.1%
With degree	24.6%	23.9%
Not in employment	40.6%	40.6%
In employment	59.5%	59.4%
Gross monthly household income (top-coded at 99th percentile)[9]	£3476.2 (1977.0)	£3411.5 (2312.9)
Living in rural area	23.3%	26.9%
Living in urban area	76.8%	73.1%
Without long-standing mental/physical illness	64.8%	64.0%
With long-standing mental/physical illness	35.2%	36.0%

Note: T1 = Time 1, Wave 2 (2010–2012). T2 = Time 2, Wave 5 (2013–2015).

Tables S5a-S6c: Non-recursive instrumental–variable models

Table S5a: Non-recursive instrumental-variable model for arts participation and mental distress (*N* = 17,927)						
	Arts participation, T2			Mental distress, T2		
	Standardised Coef.	95%CI	p-value	Standardised Coef.	95%CI	p-value
**Endogenous predictors, T2**						
Arts participation				–0.01	–0.04, 0.03	0.769
Mental distress	–0.00	–0.04, 0.03	0.873			
**Auxiliary instrumental variables, T1**						
Arts participation	0.43	0.42, 0.45	< 0.001			
Mental distress				0.43	0.41, 0.44	< 0.001
**Model implied instrumental variables, T1**						
Average regional rate of arts participation	0.03	0.02, 0.05	< 0.001			
Average regional rate of public library visits	–0.00	–0.02, 0.02	0.909			
Knowing someone who passed away				–0.01	–0.02, 0.01	0.368
Diagnosed clinical depression				0.01	–0.01, 0.03	0.149
**Variances**						
ε_1 –>	0.75	0.73, 0.76				
ε_2 –>	0.77	0.75, 0.78				
ε_1 <–> ε_2	–0.02	–0.07, 0.04	0.520			
**Fit statistic**						
SRMR	0.000					
CD	0.426					
Note: T1 = Time 1, Wave 2. T2 = Time 2, Wave 5. SRMR=Standardised root mean squared residual. CD = Coefficient of determination.						

Table S5b: Non-recursive instrumental-variable model for cultural attendance and mental distress (*N* = 17,927)

	Cultural attendance, T2			Mental distress, T2		
	Standardised Coef.	95%CI	p-value	Standardised Coef.	95%CI	p-value
**Endogenous predictors, T2**						
Cultural attendance				–0.06	–0.09, –0.03	< 0.001
Mental distress	–0.08	–0.11, –0.05	< 0.001			
**Auxiliary instrumental variables, T1**						
Cultural attendance	0.50	0.48, 0.51	< 0.001			
Mental distress				0.42	0.40, 0.44	< 0.001
**Model implied instrumental variables, T1**						
Average regional rate of cultural attendance	0.03	0.02, 0.05	< 0.001			
Average regional rate of public library visits	–0.01	–0.03, 0.01	0.446			
Knowing someone who passed away				–0.01	–0.02, 0.01	0.337
Diagnosed clinical depression				0.01	–0.01, 0.03	0.153
**Variances**						
ε_1 –>	0.64	0.63, 0.65				
ε_2 –>	0.76	0.75, 0.78				
ε_1 <–> ε_2	0.09	0.04, 0.14	< 0.001			
**Fit statistic**						
SRMR	0.001					
CD	0.511					

Note: T1 = Time 1, Wave 2. T2 = Time 2, Wave 5. SRMR=Standardised root mean squared residual. CD = Coefficient of determination.

Table S5c: Non-recursive instrumental-variable model for heritage visits and mental distress (*N* = 17,927)						
	Heritage visits, T2			Mental distress, T2		
	Standardised Coef.	95%CI	p-value	Standardised Coef.	95%CI	p-value
**Endogenous predictors, T2**						
Heritage visits				–0.05	–0.08, –0.02	0.004
Mental distress	–0.06	–0.09, –0.03	< 0.001			
**Auxiliary instrumental variables, T1**						
Heritage visits	0.46	0.44, 0.47	< 0.001			
Mental distress				0.42	0.41, 0.44	< 0.001
**Model implied instrumental variables, T1**						
Average regional rate of heritage visits	0.04	0.03, 0.05	< 0.001			
Average regional rate of public library visits	0.02	0.01, 0.04	0.003			
Knowing someone who passed away				–0.01	–0.02, 0.01	0.403
Diagnosed clinical depression				0.01	–0.01, 0.03	0.154
**Variances**						
ε_1 –>	0.68	0.67, 0.69				
ε_2 –>	0.76	0.75, 0.78				
ε_1 <–> ε_2	0.08	0.03, 0.12	0.001			
**Fit statistic**						
SRMR	0.001					
CD	0.478					
Note: T1 = Time 1, Wave 2. T2 = Time 2, Wave 5. SRMR=Standardised root mean squared residual. CD = Coefficient of determination.						

Table S6a: Non-recursive instrumental-variable model for arts participation and mental well-being (*N* = 17,927)

	Arts participation, T2			Mental well-being, T2		
	Standardised Coef.	95%CI	p-value	Standardised Coef.	95%CI	p-value
**Endogenous predictors, T2**						
Arts participation				0.04	0.01, 0.08	0.009
Mental well-being	0.05	0.02, 0.08	0.002			
**Auxiliary instrumental variables, T1**						
Arts participation	0.43	0.42, 0.45	< 0.001			
Mental well-being				0.54	0.53, 0.56	< 0.001
**Model implied instrumental variables, T1**						
Average regional rate of arts participation	0.03	0.02, 0.05	< 0.001			
Average regional rate of public library visits	–0.00	–0.02, 0.02	0.943			
Knowing someone who passed away				0.02	0.01, 0.03	0.001
Diagnosed clinical depression				–0.01	–0.03, 0.01	0.313
**Variances**						
ε_1 –>	0.75	0.73, 0.76				
ε_2 –>	0.62	0.61, 0.64				
ε_1 <–> ε_2	–0.05	–0.10, –0.00	0.043			
**Fit statistic**						
SRMR	0.001					
CD	0.533					

Note: T1 = Time 1, Wave 2. T2 = Time 2, Wave 5. SRMR=Standardised root mean squared residual. CD = Coefficient of determination.

Table S6b: Non-recursive instrumental-variable model for cultural attendance and mental well-being (*N* = 17,927)						
	Cultural attendance, T2			Mental well-being, T2		
	Standardised Coef.	95%CI	p-value	Standardised Coef.	95%CI	p-value
Endogenous predictors, T2						
Cultural attendance				0.08	0.06, 0.11	< 0.001
Mental well-being	0.11	0.09, 0.14	< 0.001			
Auxiliary instrumental variables, T1						
Cultural attendance	0.49	0.48, 0.51	< 0.001			
Mental well-being				0.54	0.52, 0.55	< 0.001
Model implied instrumental variables, T1						
Average regional rate of cultural attendance	0.03	0.02, 0.05	< 0.001			
Average regional rate of public library visits	–0.01	–0.02, 0.01	0.504			
Knowing someone who passed away				0.02	0.01, 0.03	< 0.001
Diagnosed clinical depression				–0.01	–0.03, 0.01	0.327
Variances						
ε_1 –>	0.64	0.63, 0.65				
ε_2 –>	0.62	0.61, 0.64				
ε_1 <–> ε_2	–0.14	–0.18, –0.09	< 0.001			
Fit statistic						
SRMR	0.001					
CD	0.600					
Note: T1 = Time 1, Wave 2. T2 = Time 2, Wave 5. SRMR=Standardised root mean squared residual. CD = Coefficient of determination.						

Table S6c: Non-recursive instrumental-variable model for heritage visits and mental well-being (*N* = 17,927)						
	Heritage visits, T2			Mental well-being, T2		
	Standardised Coef.	95%CI	p-value	Standardised Coef.	95%CI	p-value
**Endogenous predictors, T2**						
Heritage visits				0.08	0.05, 0.11	< 0.001
Mental well-being	0.08	0.06, 0.11	< 0.001			
**Auxiliary instrumental variables, T1**						
Heritage visits	0.45	0.44, 0.47	< 0.001			
Mental well-being				0.54	0.52, 0.55	< 0.001
**Model implied instrumental variables, T1**						
Average regional rate of heritage visits	0.04	0.03, 0.05	< 0.001			
Average regional rate of public library visits	0.02	0.01, 0.04	0.003			
Knowing someone who passed away				0.02	0.01, 0.03	0.001
Diagnosed clinical depression				–0.01	–0.03, 0.01	0.324
**Variances**						
ε_1 –>	0.68	0.67, 0.69				
ε_2 –>	0.62	0.61, 0.64				
ε_1 <–> ε_2	–0.11	–0.16, –0.07	< 0.001			
**Fit statistic**						
SRMR	0.001					
CD	0.572					
Note: T1 = Time 1, Wave 2. T2 = Time 2, Wave 5. SRMR=Standardised root mean squared residual. CD = Coefficient of determination.						

Tables S7a-S8c: Cross–lagged models

Table S7a: Cross-lagged structural equation model for arts participation and mental distress (*N* = 17,927)						
	Arts participation, T2			Mental distress, T2		
	Standardised Coef.	95%CI	p-value	Standardised Coef.	95%CI	p-value
Arts participation, T1	0.43	0.42, 0.45	< 0.001	–0.00	–0.02, 0.01	0.770
Mental distress, T1	–0.00	–0.02, 0.01	0.859	0.43	0.41, 0.44	< 0.001
**Model implied instrumental variables, T1**						
Average regional rate of arts participation	0.03	0.02, 0.05	< 0.001			
Average regional rate of public library visits	–0.00	–0.02, 0.02	< 0.001			
Knowing someone who passed away				–0.01	–0.02, 0.01	0.368
Diagnosed clinical depression				0.01	–0.01, 0.03	0.148
**Variances**						
ε_1 –>	0.75	0.74, 0.76				
ε_2 –>	0.77	0.75, 0.78				
ε_1 <–> ε_2	–0.03	–0.04, –0.01	0.003			
**Fit statistic**						
SRMR	0.000					
CD	0.426					
Note: T1 = Time 1, Wave 2. T2 = Time 2, Wave 5. SRMR=Standardised root mean squared residual. CD = Coefficient of determination.						

Table S7b: Cross-lagged structural equation model for cultural attendance and mental distress (*N* = 17,927)						
	Cultural attendance, T2			Mental distress, T2		
	Standardised Coef.	95%CI	p-value	Standardised Coef.	95%CI	p-value
Cultural attendance, T1	0.50	0.49, 0.51	< 0.001	–0.03	–0.05, –0.01	< 0.001
Mental distress, T1	–0.03	–0.05, –0.02	< 0.001	0.42	0.41, 0.44	< 0.001
**Model implied instrumental variables, T1**						
Average regional rate of cultural attendance	0.04	0.02, 0.05	< 0.001			
Average regional rate of public library visits	–0.01	–0.03, 0.01	0.436			
Knowing someone who passed away				–0.01	–0.02, 0.01	0.414
Diagnosed clinical depression				0.01	–0.01, 0.03	0.151
**Variances**						
ε_1 –>	0.64	0.63, 0.65				
ε_2 –>	0.76	0.75, 0.78				
ε_1 <–> ε_2	–0.05	–0.07, –0.04	< 0.001			
**Fit statistic**						
SRMR	0.001					
CD	0.506					
Note: T1 = Time 1, Wave 2. T2 = Time 2, Wave 5. SRMR=Standardised root mean squared residual. CD = Coefficient of determination.						

Table S7c: Cross-lagged structural equation model for heritage visits and mental distress (*N* = 17,927)						
	Heritage visits, T2			Mental distress, T2		
	Standardised Coef.	95%CI	p-value	Standardised Coef.	95%CI	p-value
Heritage visits, T1	0.46	0.44, 0.48	< 0.001	–0.02	–0.04, –0.01	0.004
Mental distress, T1	–0.03	–0.04, –0.01	< 0.001	0.42	0.41, 0.44	< 0.001
**Model implied instrumental variables, T1**						
Average regional rate of heritage visits	0.04	0.03, 0.05	< 0.001			
Average regional rate of public library visits	0.02	0.01, 0.04	0.003			
Knowing someone who passed away				–0.01	–0.02, 0.01	0.367
Diagnosed clinical depression				0.01	–0.01, 0.03	< 0.001
**Variances**						
ε_1 –>	0.68	0.67, 0.69				
ε_2 –>	0.77	0.75, 0.78				
ε_1 <–> ε_2	–0.03	–0.05, –0.01	< 0.001			
**Fit statistic**						
SRMR	0.001					
CD	0.475					
Note: T1 = Time 1, Wave 2. T2 = Time 2, Wave 5. SRMR=Standardised root mean squared residual. CD = Coefficient of determination.						

Table S8a: Cross-lagged structural equation model for arts participation and mental well-being (N = 17,927)						
	Arts participation, T2			Mental well-being, T2		
	Standardised Coef.	95%CI	p-value	Standardised Coef.	95%CI	p-value
Arts participation, T1	0.43	0.42, 0.45	< 0.001	0.02	0.00, 0.03	0.010
Mental well-being, T1	0.03	0.01, 0.04	0.002	0.55	0.53, 0.56	< 0.001
**Model implied instrumental variables, T1**						
Average regional rate of arts participation	0.03	0.02, 0.05	< 0.001			
Average regional rate of public library visits	–0.00	–0.02, 0.02	0.929			
Knowing someone who passed away				0.02	0.01, 0.03	0.001
Diagnosed clinical depression				–0.01	–0.03, 0.01	0.296
**Variances**						
ε_1 –>	0.75	0.73, 0.76				
ε_2 –>	0.62	0.61, 0.64				
ε_1 <–> ε_2	0.04	0.02, 0.06	< 0.001			
**Fit statistic**						
SRMR	0.001					
CD	0.531					

Note: T1 = Time 1, Wave 2. T2 = Time 2, Wave 5. SRMR=Standardised root mean squared residual. CD = Coefficient of determination.

Table S8b: Cross-lagged structural equation model for cultural attendance and mental well-being (*N* = 17,927)						
	Cultural attendance, T2			Mental well-being, T2		
	Standardised Coef.	95%CI	p-value	Standardised Coef.	95%CI	p-value
Cultural attendance, T1	0.50	0.48, 0.51	< 0.001	0.04	0.03, 0.06	< 0.001
Mental well-being, T1	0.06	0.05, 0.08	< 0.001	0.54	0.53, 0.56	< 0.001
Model implied instrumental variables, T1						
Average regional rate of cultural attendance	0.03	0.02, 0.05	< 0.001			
Average regional rate of public library visits	–0.01	–0.02, 0.01	0.511			
Knowing someone who passed away				0.02	0.01, 0.03	0.001
Diagnosed clinical depression				–0.01	–0.03, 0.01	0.295
Variances						
ε_1 –>	0.64	0.63, 0.65				
ε_2 –>	0.62	0.61, 0.64				
ε_1 <–> ε_2	0.06	0.04, 0.08	< 0.001			
Fit statistic						
SRMR	0.001					
CD	0.592					
Note: T1 = Time 1, Wave 2. T2 = Time 2, Wave 5. SRMR=Standardised root mean squared residual. CD = Coefficient of determination.						

Table S8c: Cross-lagged structural equation model for heritage visits and mental well-being (N = 17,927)						
	Heritage visits, T2			Mental well-being, T2		
	Standardised Coef.	95%CI	p-value	Standardised Coef.	95%CI	p-value
Heritage visits, T1	0.46	0.44, 0.47	< 0.001	0.04	0.02, 0.05	< 0.001
Mental well-being, T1	0.05	0.03, 0.06	< 0.001	0.54	0.53, 0.56	< 0.001
Model implied instrumental variables, T1						
Average regional rate of heritage visits	0.04	0.03, 0.05	< 0.001			
Average regional rate of public library visits	0.02	0.01, 0.04	0.003			
Knowing someone who passed away				0.02	0.01, 0.03	0.001
Diagnosed clinical depression				–0.01	–0.03, 0.01	0.311
Variances						
ε_1 –>	0.68	0.67, 0.69				
ε_2 –>	0.62	0.61, 0.64				
ε_1 <–> ε_2	0.05	0.04, 0.07	< 0.001			
Fit statistic						
SRMR	0.001					
CD	0.566					
Note: T1 = Time 1, Wave 2. T2 = Time 2, Wave 5. SRMR=Standardised root mean squared residual. CD = Coefficient of determination.						
